# Success and safety of deep sedation as a primary anaesthetic approach for transvenous lead extraction: a retrospective analysis

**DOI:** 10.1038/s41598-023-50372-1

**Published:** 2023-12-27

**Authors:** Fabian Schiedat, Julian Fischer, Assem Aweimer, Dominik Schöne, Ibrahim El-Battrawy, Christoph Hanefeld, Andreas Mügge, Axel Kloppe

**Affiliations:** 1 Department of Cardiology and Angiology at University Hospital Bergmannsheil Bochum of the Ruhr-University, Bochum, Germany; 2 Department of Cardiology and Angiology at Marienhospital Gelsenkirchen, Academic Hospital of the Ruhr University Bochum, Gelsenkirchen, Germany; 3 Department of Molecular and Experimental Cardiology, Institut Für Forschung Und Lehre (IFL), Ruhr-University, Bochum, Germany; 4 Department of Cardiology at Katholische Kliniken Bochum of the Ruhr University, Bochum, Germany

**Keywords:** Cardiac device therapy, Outcomes research

## Abstract

There is a rising number in complications associated with more cardiac electrical devices implanted (CIED). Infection and lead dysfunction are reasons to perform transvenous lead extraction. An ideal anaesthetic approach has not been described yet. Most centres use general anaesthesia, but there is a lack in studies looking into deep sedation (DS) as an anaesthetic approach. We report our retrospective experience for a large number of procedures performed with deep sedation as a primary approach. Extraction procedures performed between 2011 and 2018 in our electrophysiology laboratory have been included retrospectively. We began by applying a bolus injection of piritramide followed by midazolam as primary medication and would add etomidate if necessary. For extraction of leads a stepwise approach with careful traction, locking stylets, dilator sheaths, mechanical rotating sheaths and if needed snares and baskets has been used. A total of 780 leads in 463 patients (age 69.9 ± 12.3, 31.3% female) were extracted. Deep sedation was successful in 97.8% of patients. Piritramide was used as the main analgesic medication (98.5%) and midazolam as the main sedative (94.2%). Additional etomidate was administered in 15.1% of cases. In 2.2% of patients a conversion to general anaesthesia was required as adequate level of DS was not achieved before starting the procedure. Sedation related complications occurred in 1.1% (n = 5) of patients without sequalae. Deep sedation with piritramide, midazolam and if needed additional etomidate is a safe and feasible strategy for transvenous lead extraction.

## Introduction

There is a rising number in complications associated with more cardiac electrical devices implanted (CIED). The main reasons to perform transvenous lead extraction (TLE) are infections (2/3 of which are pocket associated) and lead dysfunctions^1^. In addition to this, there is also an increasing number of extractions due to a high burden of electrodes (> 5 electrodes via superior vena cava) and relevant tricuspid regurgitation^2–4^. With an increasing age of patients presenting for TLE, there are increasing comorbidities and lead dwelling time is increasing. According to the heart rhythm society (HRS) guidelines, most centres perform TLE under general anaesthesia (GA) to minimize patient discomfort, facilitate the use of transoesophageal echocardiography (TOE) during the procedure and allow the anaesthesia team to prioritize on resuscitative measures during major intraprocedural complications^4^. In a registry of the European Heart Rhythm Association (EHRA), the European lead extraction study (ELECTRa), a homogenous distribution of general anaesthesia (39%), local anaesthesia (31%) and deep sedation (31%) has been reported^1^. To this date however there has only been one large centre to report their experience with deep sedation (DS) performed with fentanyl and propofol in a large cohort^5^. Performing DS in cardiac procedures safely and successful has been reported for device implantations, ablation of arrhythmias and even for more complex procedures as transvalvular valve repair (TVR) and transcatheter aortic valve replacement (TAVR)^6–9^. In the present work, we assessed whether DS is a safe and feasible primary sedation strategy for TLE.

## Methods

### Study population

463 consecutive patients over a period of seven years have been referred to our centre for transvenous lead extraction. We included all patients in this study retrospectively. We obtained informed consent at the time of extraction, afterwards or at time of retrospective inclusion in this study. The study protocol conforms to the ethical guidelines of the 1964 Declaration of Helsinki and its later amendments. It was approved by the local ethics committee. All patients received a 12 lead electrocardiogram (ECG), thoracic x-ray, CIED interrogation, laboratory tests and transthoracic echocardiography (TTE) before the procedure. If an infection was present, TOE was performed as well. Medical history was obtained and perioperative risk was classified according to the American Society of Anaesthesiologists (ASA, physical status classification system).

### Procedure setting and sedation

Procedures were performed in our electrophysiology (EP) laboratory. Two experienced operators were performing every procedure. The operators were experienced in intensive care and emergency medicine with advanced life support training and were trained in airway management, including difficult airways. Three nurses were present during procedure. One nurse was sterile and supporting the operation. One nurse with experience in intensive care medicine administered intravenous drugs under supervision of the operators and monitored vital signs. A third nurse was non-sterile to assist.

During the procedure an anaesthesiologist, a cardiac surgeon, a perfusionist and supporting staff were available in standby within 2 min as back-up. During the time of the procedure they had no other duties. Equipment for conversion to GA or open heart surgery was present in the EP laboratory, including a heart–lung machine. Monitoring was performed with continuous ECG, oxygen saturation and invasive blood measurements. Arterial blood gas analyses were performed once an hour and if needed more frequently. All patients received a central venous catheter before the procedure and a femoral sheath with a stiff wire advanced in any jugular, subclavian or brachiocephalic vein to enable rapid deployment of a endovascular occlusion balloon in case of superior vena cava injury. This was routinely established since introduction of the occlusion balloon in 2016. TOE was performed in patients with passive fixation right atrium (RA) or right ventricular (RV) electrodes. In all other cases TTE with probe placed in two sterile sheaths and sterile acoustic gel was available and focused echocardiographic evaluation for pericardial effusion was performed immediately after extraction of every lead. In all patients DS was the primary approach as DS is the standard approach for all device procedures at our centre and no guidelines existed at the time of procedure that recommended otherwise. Sedation was initiated by a bolus of piritramide (7.5 mg) and midazolam (2.5 mg) according to hospital standards. Additional bolus applications were administered to achieve a level of deeper sedation or during the procedure to maintain DS if necessary (3.75 mg piritramide and/or 1.5 mg midazolam). In cases of a history of allergic reaction to these drugs, sufentanil bolus (0.1 µg/kg) was given instead of piritramide and propofol bolus (0.5 mg/kg) with continuous infusion (3.5 mg/kg/h) instead of midazolam. If a level of deep sedation was not achieved after a total dose of 8.5 mg midazolam, etomidate bolus (2 mg, uptitration in 1 mg steps) was added to achieve a level of DS. DS was classified according to the ASA as breathing spontaneously with unresponsiveness to vocal stimuli and tolerating an oropharyngeal airway^10^. There were no restrictions for DS as a primary sedative strategy.

Patients received continuous norepinephrine if mean arterial pressure (MAP) dropped below 60 mmHg. Oxygen was applied via nose canula to maintain a peripheral saturation level > 90%. If peripheral oxygen saturation dropped below 90% head and neck position was optimised, jaw-thrust manoeuvre was performed and a guedel tube was introduced over which oxygen was applied. If these manoeuvres failed patients were ventilated by face mask and endotracheal intubation was performed by an anaesthesiologist if necessary. If an intubation was necessary, the procedure was continued with GA. In cases of serious complications the anaesthesiology team, and if necessary cardiac thoracic team, is informed immediately. As both physicians performing the procedure are experienced in intensive care medicine, one physician took over airway and medication management during serious complications until the anaesthesiology team arrived. During this time the other physician performed all necessary measures to handle the complication. Standard operating procedures for such cases are in place at our centre.

If a level of DS was achieved 1% Mepivacaine was administered at the surgical location.

### Lead extractions

Pacemaker dependent patients received a temporary right ventricular pacemaker lead via an additional femoral venous access and if re-implantation needed to be postponed, a transcutaneous screw-in right ventricular lead was inserted and connected to an external pacemaker after extraction. After application of mepivacaine the device pocket was opened and the leads including their sleeves were exposed. A standard stylet was inserted and simple traction was performed. If not successful a locking stylet was inserted and a stepwise approach with traction, mechanical polypropylene sheaths, mechanical rotating sheaths and a snare was used. This stepwise approach with a progressive invasive strategy was not influenced by the sedation method and will be reported in detail elsewhere. Success and complications of lead extractions were recorded according to the Heart Rhythm Society and EHRA guidelines for TLE. Procedure time (skin incision time to skin suture) has been documented.

### Data collection

Data has been collected continuously, analysed retrospectively and checked again afterwards with clinical information system.

### Statistical analysis

All statistical analysis was performed using IBM SPSS Statistics version 24.0.0 on mac.

Categorial variables were expressed as frequencies and percentages (normal distribution) or median and interquartile range (non-normal distribution). Continuous variables were stated as mean ± standard deviation. All variables listed in Table 1 were evaluated for DS related complications in a univariate cox proportional hazard model. A p < 0.05 was considered statistically significant.

**Table 1:** Baseline characteristics and acute measurement results (n = 463).

Characteristics	Value
Age (years)	69.9 ± 12.3
Sex, female n (%)	145 (31.3)
BMI (kg/m^2^)	27.5 ± 5.6
Weight (kg)	82.4 ± 19.5
Renal Insufficiency, n (%)	168 (36.3)
I, n (%)	8 (1.7)
II, n (%)	36 (7.8)
III, n (%)	97 (21.0)
IV, n (%)	14 (3.0)
V with dialysis, n (%)	14 (3.0)
Arterial hypertension, n (%)	416 (89.9)
Diabetes mellitus, n (%)	145 (31.1)
COPD, n (%)	95 (20.5)
Coronary artery disease, n (%)	253 (54.6)
Heart failure, n (%)	254 (54.9)
NYHA III/IV, n (%)	154 (33.3)
History of ventricular arrhythmia, n (%)	150 (32.4)
LV-EF (%)	45.3 ± 12.6
ASA III- IV, n (%)	128 (27.6)
System	
Pacemaker, n (%)	205 (44.4)
ICD, n (%)	254 (54.8)
CCM, n (%)	4 (0.8)
Left sided system, n (%)	395 (85.3)
Reason for extraction	
Infection, n (%)	156 (33.7)
Lead malfunction or dislocation, n (%)	244 (52.7)
Lead perforation, n (%)	45 (9.7)
System upgrade, n (%)	18 (3.9)
Pacemaker leads	447
Pace/Sense leads	32
ICD leads	244
Single coil	201
Dual coil	43
Fixation mode	
Screw (active)	565
Passive	126
CS passive	89

### Ethical approval

The study protocol conforms to the ethical guidelines of the 1964 Declaration of Helsinki and its later amendments. It was approved by the ethics committee of the Ruhr University Bochum (Register 18-6516).

### Informed consent

Informed consent was obtained from all subjects involved in this study.

## Results

### Baseline characteristics

A total of 463 patients who underwent total lead extraction of 780 leads between January 2011 and august 2018 were retrospectively included in this study. All patients who received a lead extraction at our centre during that time were included in this study. There were no patients during the time that opted for GA as a primary approach. Mean age was 69.9 ± 12.3 years and 31.3% (n = 145) were female. Left ventricular ejection fraction (LV-EF) was 45.3 ± 12.6%. Main reasons for extraction were infection and lead dysfunction. Baseline patient characteristics are listed in Table 1.

### Procedure and sedation

Procedure time was 103.4 ± 68.8 min and 98.5% (n = 456) received an initial bolus of 90.0 µg/kg (86.8–93.2) piritramide. Shortly after a midazolam bolus of 0.031 mg/kg (0.027–0.035) was administered in 94.2% (n = 436) of all patients. A bolus of sufentanil was administered in 1.5% (n = 7). Propofol was given in 5.8% (n = 27). Etomidate was given additionally to midazolam in 15.1% (n = 69). Deep sedation was successful in 97.8% (n = 453), with 2.2% (n = 10) being converted to GA as a level of DS was not reached before starting the procedure. We did not identify any variables associated with need to conversion to GA. Procedural and sedations characteristics are summarized in Table 2.

**Table 2:** Procedural characteristics and outcome.

Characteristics	Value
Procedure time (min)	103.4 ± 68.8
Lead dwelling time, years	5.4 ± 4.9
Leads per patient for extraction	
1, n (%)	252 (54.4)
2, n (%)	124 (26.8)
3, n (%)	70 (15.1)
4, n (%)	16 (3.5)
5, n	0
6, n (%)	1 (0.2)
Extraction completely successful by tool	
Lead locking stylet and traction	458 (58.7)
Mechanical unpowered sheaths	207 (26.5)
Mechanical rotating sheaths	25 (3.2)
Snare or basket	36 (4.6)
Total success rate, n (%)	726 (93.1)
Clinical success rate, n (%)	734 (94.1)
Screw in Electrode until Re-implant, n (%)	32 (6.9)
Minimum MAP (mmHg)	72.8 (69–75)
Minimum oxygen saturation (%)	94.8 (93–96)
Peak oxygen flow (l/min)	2.23 (2.1–2.4)
Transesophageal Echocardiography during procedure, n(%)	76 (16.4)
Piritramide administered, n (%)	456 (98.5)
Piritramide initial bolus dose (µg/kg)	90.0 (86.8–93.2)
Additional bolus, n (%)	201 (44.1)
Total dose	115.2 (107.9–122.2)
Midazolam administered, n (%)	436 (94.2)
Midazolam initial bolus dose, (mg/kg)	0.031 (0.027–0.035)
Additional midazolam bolus, n (%)	226 (51.8)
Total dose, (mg/kg)	0.046 (0.030–0.052)
Propofol infusion, n (%)	27 (5.8%)
As primary anesthetic, n (%)	27 (5.8%)
As addition to midazolam, n (%)	0
Total dose (mg/kg)	4.1 (3.4–4.7)
Etomidate additionally to midazolam, n (%)	69 (15,1%)
Etomidate initial dose (mg/kg)	0.078 (0.069–0.088)
Additional bolus	18 (26.1)
Total dose (mg/kg)	0.091 (0.082–0.098)
Requirement of vasopressor, n (%)	2 (0.4)
Hypoxia requiring	1 (0.2%)
Hypercapnia requiring non-invasive CPAP, n (%)	2 (0.4)
New postoperative Delir	0 (0%)

### Success rate and complications

Of a total of 1025 leads in 463 patients, 780 leads were identified for extraction. Mean lead dwelling time was 5.4 ± 4.9 years. Of these n = 447 were pacemaker leads and n = 244 were defibrillator leads. Total success rate was 93.1% (n = 726/780) and clinical success rate was 94.1% (n = 734/780). Major intraprocedural complications occurred in n = 2 (0.4%). None of which were associated with DS. A total of 36 minor intraprocedural complications occurred in n = 30 (6.4%) patients. DS related minor intraprocedural complications occurred in n = 5 patients (1.1%). Of these two patients (0.4%) experienced a drop in MAP with the necessity to administer continuous norepinephrine to maintain a MAP of > 60 mmHg, one patient (0.2%) experienced hypoxia and was intubated with conversion to general anesthesia without further short- or long-term complications associated to hypoxia or intubation. No other patients with a minor complication required conversion to GA. And two patients (0.4%) experienced hypercapnia at the end of the procedure and were treated with non-invasive continuous positive airway pressure (CPAP) ventilation for up to 2 h after the procedure without a sequalae. There were no additional DS related postprocedural complications (e.g. late onset hypotension or late onset hypercapnia). There were no DS associated complications associated with etomidate. We did not identify any parameters in a univariate cox proportional hazard model associated with DS related intraprocedural complications.

## Discussion

We demonstrated good safety and feasibility for DS in 463 patients undergoing TLE. To our knowledge this is the first study to show an approach with piritramide and propofol as the primary anaesthetic medication. Several studies were able to show that performing simple and complex device implantations under DS is safe^7,11^. According to guidelines an approach with GA is recommended for TLE to increase comfort and facilitate TOE^4^. With the patient cohort presenting for TLE continuously being older, risks of GA need to be considered^12^. For complex cardiac procedures however, good data exists that DS and GA are without significant differences in terms of safety^9^. Our retrospective analysis was therefore performed to investigate whether it is safe to perform DS for TLE as well, as no guidelines existed at the time of the procedures regarding the anaesthetic approach for these procedures. Resources for cardiac procedures with an anaesthesiology teams present during the whole procedure are scarce at our centre, other centres in the region and worldwide, hence offering possible advantages for DS in these procedures that often have to be performed on short notice^13^. For procedures that require TOE during a procedure, DS has been reported to be safe and feasible^6^. Although nowadays TOE is recommended in TLE, we did not use it routinely in every case included in this study as no recommendations existed to routinely perform TOE or intracardiac echocardiography (ICE) during TLE until September 2017 in the US and July 2018 in Europe^3,4^. In addition there is no comparative data that favours routine use of TOE in TLE and there are complications that can derive from TOE^4,14,15^. If TLE is performed using laser lead removal, which is associated with a higher rate in major complications, TOE with prompt diagnosis of these complications can be helpful^16^. Our strategy was to use TOE routinely in cases with passive fixation RA and RV leads. Use of TOE was not associated with failure of DS or DS associated complications in our study. We think that according to previous data and our study, DS would be feasible for centres with routine use of TOE as well. Since beginning of 2019 we implemented routine use of TOE or ICE according to guidelines as well^4^.

So far only Bode et al. reported their experience with DS in TLE in a large cohort^5^. They reported DS to be safe with a high success rate^1^. In comparison to our study however, they primarily administered Fentanyl and Propofol. They reported higher rates of patients experiencing hypoxia or hypercapnia related events compared to our study (2.3 vs. 0.6%). They were able to show an association for Fentanyl, dose of Fentanyl and patients requiring further support during the procedure^5^. In our study we used Piritramide as a an opioid. Piritramide is widely used in Germany and other European countries for complex and long lasting cardiac procedures like TVR, TAVR and complex device implantations with good safety outcomes^6,17^. These studies were also able to show lower needs for inotropic medication and fewer patients requiring a postprocedural intensive care unit (ICU) stay for DS compared to GA^16^. For piritramide compared to fentanyl time to pain relief is longer (16.8 vs. 0.5 – 2.0 min), analgetic potency is lower (0.7–0.75 vs. 100), but its effect lasts much longer (4–6 h vs. 0.3–0.5 h), thus requiring less repetitive bolus injections during long procedures^18^. This could explain a higher rate in conversion to GA but lower rates of hypoxia or hypercapnia in our cohort. The one reported DS associated conversion to GA with intubation in our cohort was without complication according to our standard operating procedure for a case like this with The anaesthesiology team arriving within one minute. During both major complications a conversion to open heart surgery was necessary. As the complication occurred airway and medication management was performed by one operator according to our standards described in the methods section and the procedures were converted within 2 min with anaesthesia team arriving. DS did not prolong the time until conversion and hypoxia as well as conversion to GA did not impact the outcome. Compared to Bode et al., we additionally documented lower rates of patients requiring vasopressors for hypotension (11.4% vs. 0.4%)^5^. A reason for this could be the use of midazolam instead of propofol for sedation. For midazolam a lower drop in systolic and diastolic blood pressure has been described compared to propofol in other cardiac procedures^19^. For atrial fibrillation ablation persistent hypotension requiring cessation of propofol has been described for up to 14% of cases^20^. For GA compared to DS even higher rates of hypotension requiring vasopressors (1.1 ± 1.6 mg norepinephrine for GA vs. 0.2 ± 0.3 mg for DS) have been described^6^. However, for propofol compared to midazolam, a deeper level of sedation with faster onset has been reported^21^. This explains the high rate of patients requiring additional sedative medication in our study (15.1%). All patients received bolus injection of etomidate if a level of deep sedation was not achieved after administering piritramide and midazolam. Advantages of etomidate are fast onset, short duration of action and hemodynamic stability in critically ill patients^22^. Although suppression of adrenal gland function has been described especially for long-term use, there is no evidence for a higher mortality or adverse outcome after bolus injection in critical ill patients^22,23^. This approach for DS with piritramide, midazolam and if needed addition of etomidate could explain the low rate of sedation related complications in our cohort.

We performed all procedures in the EP laboratory. For this approach a similar rate of complications and mortality has been described by Franceschi et al. compared to TLE in the operating room^24^. Compared to Franceschi we experienced a lower major complication rate with a similar success rate with our routinely used progressive invasive stepwise extraction approach. This approach and its success was not influenced by the choice of sedation. Compared to data published by Kancharla et al., our cohort was at an intermediate-high risk^25^. In low, intermediate and high risk cases it can be reasonable to perform TLE in the EP laboratory with equipment present but without anaesthesiology availability within 2 min due to very low complication rates with our approach. In cases at very high risk (lead dwelling time > 10 years, passive fixation leads and in cases with indication for conversion to open chest extraction if transvenous TLE fails), it seems reasonable to perform TLE in a hybrid operation room with anaesthesiologic and cardiac surgical support present in the room.

## Limitation

In our single-center experience with a sufficient number of patients we neither compared DS with GA, nor did we compare different opioid medications or sedatives for DS. Our results therefore reflect only our experience with our standard approach. Further randomized studies are required to compare GA and DS for TLE according to risk stratification.

## Conclusion

For experienced centres with established routines for DS in complex cardiac procedures, DS appears feasible and safe for TLE in the EP laboratory. Risk stratification needs to be considered to identify patients who need further support during the procedure. Further randomized studies are required to support our results and identify patients that might require GA.

## Data Availability

All data can be made available by Fabian Schiedat upon request.

## Abbreviations

ASA: American Society of Anaesthesiologists
BMI: Body mass index
CCM: Cardiac contractility modulation
CIED: Cardiac electrical devices implanted
COPD: Chronic obstructive pulmonary disease
CPAP: Continuous positive airway pressure
CS: Coronary sinus
DS: Deep sedation
ECG: Electrocardiogram
EHRA: European heart rhythm society
EP: Electrophysiology
GA: General anaesthesia
HRS: Heart rhythm society
ICD: Implantable cardioverter defibrillator
ICE: Intracardiac echocardiography
LV-EF: Left ventricular ejection fraction
MAP: Mean atrial pressure
Min: Minutes
NYHA: New York Heart Association
RA: Right atrium
RV: Right ventricular
TAVR: Transcatheter aortic valve replacement
TLE: Transvenous lead extraction
TOE: Transoesophageal echocardiography
TTE: Transthoracic echocardiography
